# The Aflibercept-Induced MicroRNA Profile in the Vitreous of Proliferative Diabetic Retinopathy Patients Detected by Next-Generation Sequencing

**DOI:** 10.3389/fphar.2021.781276

**Published:** 2021-12-06

**Authors:** Ju Guo, Pengyi Zhou, Zhenhui Liu, Fangfang Dai, Meng Pan, Guangqi An, Jinfeng Han, Liping Du, Xuemin Jin

**Affiliations:** ^1^ Department of Ophthalmology, Henan Province Eye Hospital, Henan International Joint Research Laboratory for Ocular Immunology and Retinal Injury Repair, The First Affiliated Hospital of Zhengzhou University, Zhengzhou, China; ^2^ People’s Hospital of Zhengzhou University and Henan Eye Institute, Zhengzhou, China

**Keywords:** microRNA profile, next-generation sequencing, anti-vascular endothelial growth factor therapy, proliferative diabetic retinopathy, gene expression omnibus

## Abstract

**Purpose:** Vascular endothelial growth factor-A (VEGF-A) is an important pathogenic factor in proliferative diabetic retinopathy (PDR), and aflibercept (Eylea) is one of the widely used anti-VEGF agents. This study investigated the microRNA (miRNA) profiles in the vitreous of 5 idiopathic macular hole patients (non-diabetic controls), 5 untreated PDR patients (no-treatment group), and 5 PDR patients treated with intravitreal aflibercept injection (treatment group).

**Methods:** Next-generation sequencing was performed to determine the miRNA profiles. Deregulated miRNAs were validated with quantitative real-time PCR (qRT-PCR) in another cohort. The mRNA profile data (GSE160310) of PDR patients were retrieved from the Gene Expression Omnibus (GEO) database. The function of differentially expressed miRNAs and mRNAs was annotated by bioinformatic analysis and literature study.

**Results:** Twenty-nine miRNAs were significantly dysregulated in the three groups, of which 19,984 target mRNAs were predicted. Hsa-miR-3184-3p, hsa-miR-24-3p, and hsa-miR-197-3p were validated to be remarkably upregulated in no-treatment group versus controls, and significantly downregulated in treatment group versus no-treatment group. In the GSE160310 profile, 204 deregulated protein-coding mRNAs were identified, and finally 179 overlapped mRNAs between the 19,984 target mRNAs and 204 deregulated mRNAs were included for further analysis. Function analysis provided several roles of aflibercept-induced miRNAs, promoting the alternation of drug sensitivity or resistance-related mRNAs, and regulating critical mRNAs involved in angiogenesis and retinal fibrosis.

**Conclusion:** Hsa-miR-3184-3p, hsa-miR-24-3p, and hsa-miR-197-3p were highly expressed in PDR patients, and intravitreal aflibercept injection could reverse this alteration. Intravitreal aflibercept injection may involve in regulating cell sensitivity or resistance to drug, angiogenesis, and retinal fibrosis.

## Introduction

Diabetic retinopathy (DR) is a common complication of diabetes mellitus and the leading cause of low vision in working-aged adults (Ogurtsova et al., 2017; Solomon, Chew et al., 2017), which consists of two stages: non-proliferative diabetic retinopathy (NPDR) is the early stage characterized by the developments of microvascular changes; proliferative diabetic retinopathy (PDR) is the later stage, characterized by the formation of neovascularization and fibrous tissue. The excessive release of vascular endothelial growth factor A (VEGF-A) in the retina was a crucial regulator of PDR by promoting the retinal neovascularization. Therefore, intravitreal anti-VEGF agent injection has been widely applied to treat PDR and diabetic macular edema (DME) patients. Indeed, anti-VEGF therapy was effective at delaying the progression of PDR as one of its first-line treatment. What is more, after anti-VEGF treatment, 24 highly expressed biomarkers were reported to normalize toward an unequivocal trend in aqueous of various ocular VEGF-related conditions (Sharma et al., 2010). However, not all patients show full response to anti-VEGF therapy (Ashraf et al., 2016), and even some studies report that prolonged injections of anti-VEGF agents may aggravate retinal ischemia (Toy et al., 2016; Lee et al., 2017), attribute to the irreversible degradation of retinal neurons and retinal pigment epithelium (RPE) (Ashraf et al., 2016; Gemenetzi et al., 2017; Keir et al., 2017), and increase the incidence of retinal fibrosis and tractional retinal detachment (Hu et al., 2014). Aflibercept is a recombinant human fusion protein that works as a soluble decoy receptor that binds to VEGF family members, including VEGF-A, VEGF-B, and placental growth factor (PIGF), to block their activities. It has been demonstrated that aflibercept reduced the activation of phospholipase A2 (PLA2)/cyclooxygenase-2 (COX-2)/prostaglandin (PG) axis in human retinal pericytes subjected to high glucose to protect it from glucose-induced damage (Giurdanella et al., 2015). All of this suggests anti-VEGF treatment may trigger complex regulatory mechanisms in the intraocular environment. Thus, there is an unmet medical need of exploring it to explain different responses and prognosis after anti-VEGF treatment.

MicroRNAs (miRNAs) are a kind of small non-coding RNAs with the length of 18–22 nucleotides that negatively regulate the expression of genes post-transcriptionally by binding to their 3′-untranslated regions (UTR). Recently, miRNAs are reported to be stable and detectable in many body fluids such us blood, urine, tears, aqueous, vitreous, and saliva (Dunmire et al., 2013; Rayner and Hennessy 2013). Circulating miRNAs within apoptotic bodies, exosomes, microvesicles (Raposo and Stoorvogel 2013), or binding to protein complexes (Vickers et al., 2011), which can protect them from endogenous ribonuclease, adverse pH, and temperature, can be transported to cells to modulate the expression of mRNAs (Hergenreider et al., 2012; Monastyrskaya 2018). Therefore, profiling the circulating miRNAs in body fluids is a useful method to get knowledge of the complex regulation and expression of genes happening in many diseases, and miRNAs have become the most promising biomarkers for disease diagnosis and prognosis. There are several approaches available to detect and qualify the expression of circulating miRNAs in body fluids, such as quantitative real-time PCR (qPCR), miRNA microarrays, droplet digital PCR, nanoString, and next-generation sequencing (NGS) (Takahashi et al., 2014). NGS is a recently developed sequencing technology that can identify more than 100 million reads per sample (Nakato et al., 2013) and show its advantage at lower detectable threshold and higher sensitivity than other sequencing technologies (Wang et al., 2009). This study aims to screen out the aflibercept-induced miRNA profile by NGS and try to explore the complex regulatory mechanisms in the intraocular environment by explaining the function of aflibercept-induced miRNAs.

## Material and Methods

### Subject Enrollment and Study Population

This cross-sectional study was carried out at the First Affiliated Hospital of Zhengzhou University from June 2020 to February 2021. It was approved by the Ethics Committee of the First Affiliated Hospital of Zhengzhou University (This trial is registered on the website^1^ and its unique identifier is approved protocol number: 2021-KY-0176-002) and was conducted according to the Declaration of Helsinki. Fifty-one patients undergoing pars plana vitrectomy were included in this study. All patients were informed of the use of their samples and signed informed consent. In the screening cohort (15 eyes of 15 patients), five patients diagnosed with idiopathic macular holes were included as control group, five PDR patients who had no history of ophthalmic therapy were included as no-treatment group, and five PDR patients who were at the seventh day after intravitreal aflibercept injection were listed as treatment group. In the validation cohort (36 eyes of 36 patients), there were respectively 12 patients in the control group, no-treatment group, and treatment group.

### Inclusion Criteria and Exclusion Criteria

PDR patients were diagnosed according to the 2017 Diabetic Retinopathy Preferred Practice Pattern Guideline issued on the website^2^ by the American Academy of Ophthalmology: patients with diabetic retinopathy should be considered to be at the PDR stage if they have one of the following manifestations: neovascularization; vitreous or preretinal hemorrhage. In our research, to minimize the effect of blood mixture, only patients with severe fibrous proliferation or tractional retinal detachment were included as PDR group. IMH patients were diagnosed based on clinical manifestation, and fundus and optical coherence tomography results. For the exclusion criteria, patients with dense vitreous hemorrhage, acute or chronic infection, history of ocular treatment, ocular trauma, other ocular diseases, severe heart, liver, and kidney diseases, and infection were excluded.

### Sample Preparation

Under local anesthesia, a 3-port 23-gauge pars plana vitrectomy was performed on all patients by the same surgeon. At the onset of surgery, 1 ml vitreous humor was extracted from the core of the vitreous cavity into a syringe connected to the cutter after the infusion was closed. Before that, the testing water in the cutter tube was pushed out by the syringe. Then the sample was injected into an enzyme-free Eppendorf (EP) tube, put on ice immediately, and centrifuged for 10 min at 12,000×*g* to remove any cell debris and blood admixture. All samples were stored at −80°C for further use. To minimize bias, all samples were labeled by subject ID according to the randomization protocol, which was concealed in identical sealed envelopes, and the investigators were unaware of the group labels.

### Profiling the miRNAs in the Vitreous by NGS

Frozen vitreous samples were thawed on ice for an hour prior to centrifugation at 12,000×*g* for 5 min at 4°C. Total RNA was extracted from 500 μl vitreous using Trizol reagent (Invitrogen, Carlsbad, CA, USA). The purity and concentration of total RNA were quantified by Nano Drop ND-1000 Spectrophotometer (Thermo Fisher Scientific, MA, USA), and an Agilent 2100 bioanalyzer (Thermo Fisher Scientific) was used to assess its integrity.

Total RNA (1 µg) per sample was used to conduct miRNA sequencing library. After size fractionating by 15% denaturing polyacrylamide gel electrophoresis, the RNA mixture was eventually isolated into small RNA fragments with the length of 18–30 nt, the 3′ and 5′ ends of which were ligated with adenylated adapters annealed to unique molecular identifiers (UMIs). Then, the tagged small RNAs were retro-transcribed into single-stranded complementary DNA (cDNA) with Superscript II Reverse Transcriptase (Invitrogen, USA) and cDNAs were amplified by PCR with PCR Primer Cocktail and PCR Mix. The PCR constructs were then separated by agarose gel electrophoresis and target fragments with the size of 110–130 bp were excised and purified by QIAquick Gel Extraction Kit (QIAGEN, Valencia, CA). The final library was qualified and quantified in two methods: the quality and production were tested by real-time quantitative PCR (TaqMan Probe) and the distribution of the fragment size was checked by Agilent 2100 bioanalyzer (Thermo Fisher Scientific). Library preparations were pooled and sequenced by the BGISEQ-500 platform (BGI-Shenzhen, China).

### Bioinformatics Analysis of Sequencing Result

Bioinformatics tools were applied to extract meaningful information from raw sequencing data. After filtering adapter sequences and low-quality tags, clean tags were aligned to the reference genome and miRbase with Bowtie2^2^ (Langmead et al., 2009). Subsequently, Rfam mapping was performed to identify tags that matched rRNA, tRNA, snRNA, snoRNA, and other ncRNAs in cmsearch-tool^3^ (Nawrocki and Eddy 2013). The software miRDeep2^4^ was used to predict novel miRNAs by exploring the secondary structure (Friedlander et al., 2008). Theoretically, the UMI counts were log-normalized to calculate the absolute expression level of miRNA. Then, the target genes of miRNAs were commonly predicted by the online tools, including RNAhybrid^5^ (Kruger and Rehmsmeier 2006), miRanda^6^ (John, Enright et al., 2004), and TargetScan^7^ (Agarwal, Bell et al., 2015). Differentially expressed miRNAs (DEMIs) were analyzed by DEGseq,^8^ and only the genes (miRNAs) with the absolute value of Log2Ratio ≥1 and Q value ≤0.01 were considered as DEMIs (Wang, Feng et al., 2010). Gene Ontology (GO) enrichment and Kyoto Encyclopedia of Genes and Genomes (KEGG) pathway enrichment were performed to annotate the function of target genes using phyper, a function of R, version 3.1.2. After being corrected by Bonferroni method, *p*-value ≤0.05 was used as the threshold.

### Microarray Data Information

The microarray expression profiling data of total RNA (GSE160310) from 80 human post-mortem retinal samples based on GPL20301 Illumina HiSeq 4000 (*Homo sapiens*) were downloaded from the Gene Expression Omnibus (GEO) database,^9^ which included 20, 20, 35, and 5 samples of the four patient groups (healthy control, diabetic, NPDR, PDR), respectively. All data were acquired and applied strictly according to the publication guidelines and data access policies of GEO database. Differential expression genes were analyzed by GEO2R online tool and corrected *p*-value <0.05 and | logFC | >1.5 were regarded as the threshold.

### Validation Through qRT-PCR

The validation experiment was conducted with quantitative real-time PCR (qRT-PCR) in another cohort including 12 IMH patients, 12 PDR no-treatment patients, and 12 PDR treatment patients. Total RNA from vitreous sample was extracted according to the manufacturer’s protocol of Trizol reagent (Servicebio, Wuhan, China) with A.*C. elegans* miR-39, a custom spike-in control. Primer sequences for retrotranscription and qRT-PCR of each miRNA were designed and purchased from Servicebio (Wuhan, China). In this research, cel-miR-39-3p (forward (F), 5′-ACA​CTC​CAG​CTG​GGG​TCA​CCG​GGT​GTA​AAT​C-3′; reverse (R), 5′-CTC​AAC​TGG​TGT​CGT​GGA​GTC​GGC​AAT​TCA​GTT​GAG​CAA​GCT​GA-3′) was used as endogenous control for normalization of the miRNA expression data. Then, the RNA was reverse-transcribed into cDNA using Servicebio RT First Strand cDNA Synthesis Kit (Servicebio, Wuhan, China) and qRT-PCR was performed with 2× SYBR Green qPCR Master Mix (High ROX) (Servicebio) in accordance with the manufacturer’s instructions. Raw qRT-PCR data were analyzed using QuantStudio Real-Time PCR System software (Applied Biosystems). All data represented the average results from three independent experiments. Cycle threshold (Ct) values for each sample were converted to arbitrary absolute amounts (2^−ΔΔCT^).

### Statistical Analysis

The statistical analysis of data was conducted using IBM SPSS-25 Statistics (IBM SPSS Statistics for Windows, version 20.0; IBM Corp., Armonk, NY, USA). The categorical variables, gender and hypertension, were presented as frequency counts and percent, and were analyzed using Pearson’s χ^2^ or Fisher’s exact test. The continuous variables were summarized as means ± SDs and medians with ranges or quartiles, and one-way ANOVA, Student t-test, or non-parametric testing, including the Kruskal-Wallis test, were conducted for their analysis. A *p*-value <0.05 indicated statistical significance.

## Result

### Baseline Characteristics of the Patients

A total of 51 patients were eligible in the study (26 males, 25 females, average age 65.65 ± 5.56), the baseline characteristics of which are presented in Table 1. All raw clinical data was listed in Supplementary File S1, Table 1. As shown in Table 1, there was no significant difference in age or gender distribution between PDR patients and controls in any of the three groups (*p* > 0.05). In terms of general condition parameters, including hypertension, diabetes duration, and HbA1c, no significant differences of them were observed between all groups in both screening cohort and validation cohort (*p* > 0.05), except for the comparison of HbA1c between control group and PDR patients.

**TABLE 1:** Demographic and clinical data of the study groups

	Screening cohort			*p*	Validation cohort			*p*
	Control	No-treatment group	Treatment group		Control	No-treatment group	Treatment group	
	(*n* = 5)	(*n* = 5)	(*n* = 5)		(*n* = 12)	(*n* = 12)	(*n* = 12)	
Age (years)	64.20 ± 3.01	65.20 ± 2.13	66.2 ± 2.06	0.85^a^	66.75 ± 1.80	64.00 ± 1.53	66.75 ± 1.70	0.34^a^
Gender								
Male	3	3	2	0.77^b^	6	6	6	1^b^
Female	2	2	3		6	6	6	
Hypertension, n%	0, 0	1, 20	1, 20	0.29^b^	0, 0	3, 25	4, 33.3	0.1^b^
Diabetes duration	NA	11.3 ± 1.6	11.5 ± 1.1	0.68^a^	NA	13.3 ± 1.3	13.3 ± 1.0	0.98^a^
HbA1c (%)	4.50 ± 0.29	9.7 ± 0.7	9.3 ± 0.9	0.01/0.75^a^	4.41 ± 0.15	8.4 ± 0.7	8.2 ± 0.5	0.00/0.44^a^

a Kruskal–Wallis test.

b Chi-square test.

### MiRNA Profiling in the Screening Cohort

**FIGURE 1:**
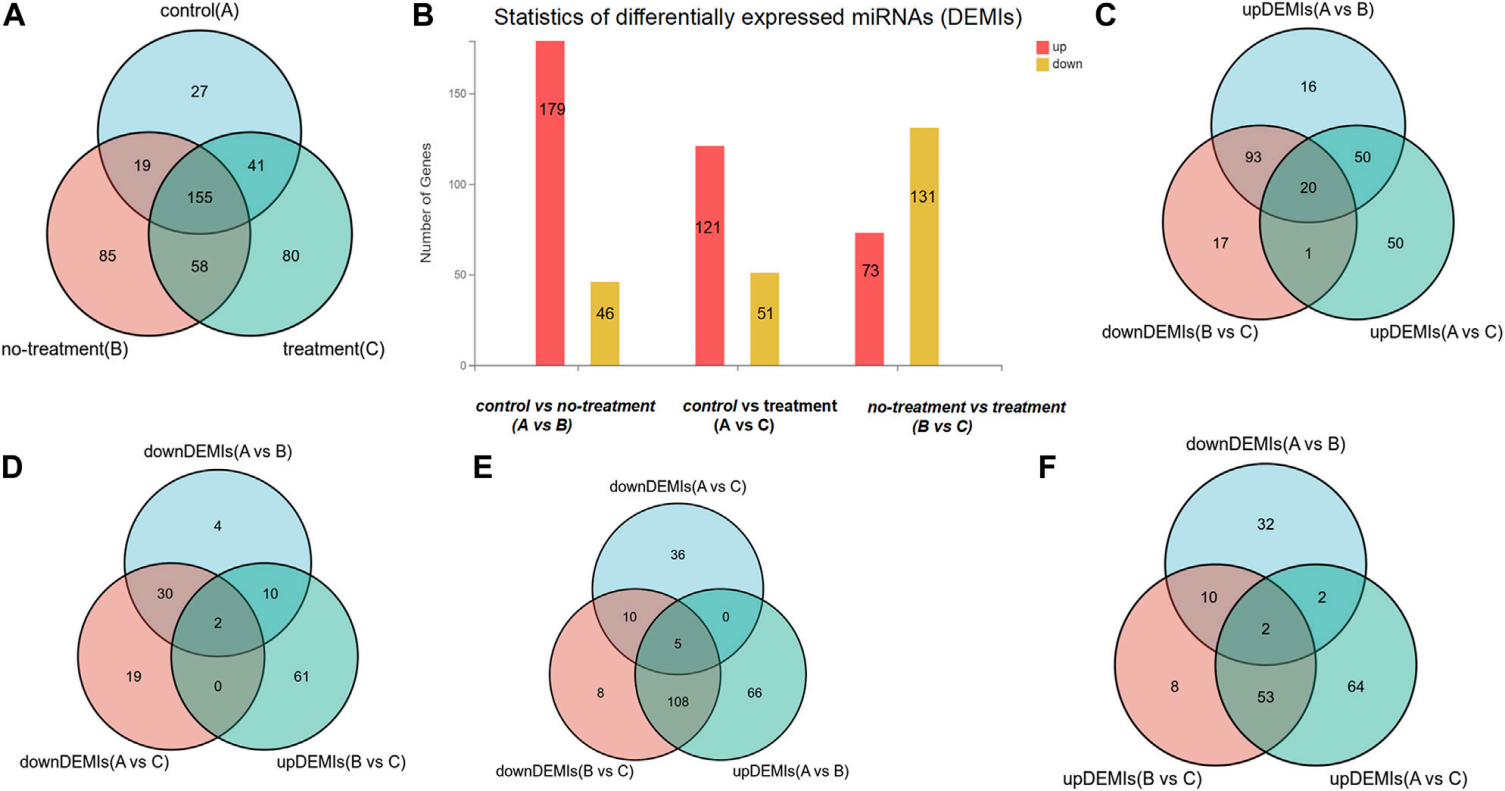
The distribution of differentially expressed miRNAs (DEMIs). **(A)** Venn diagram shows there are respectively 242, 317, and 334 DEMIs in control, treatment, and no-treatment groups. **(B)** Bar graph reveals the statistic of DEMIs, respectively 179 up-DEMIs and 46 down-DEMIs, 121 up-DEMIs and 51 down-DEMIs, and 73 up-DEMIs and 131 down-DEMIs in the comparison group of control vs. no-treatment, control vs. treatment, and no-treatment vs. treatment, which was encoded as A vs. B, A vs. C, and B vs. C. **(C)** Venn diagram screened out 20 miRNAs commonly existed in the comparison group of up-DEMIs (A vs. B), up-DEMIs (A vs. C), and down-DEMIs (B vs. C). **(D)** Venn diagram shows two miRNAs that commonly existed in the comparison group of down-DEMIs (A vs. B), down-DEMIs (A vs. C), and up-DEMIs (B vs. C). **(E)** Venn diagram shows five miRNAs that commonly existed in the comparison group of up-DEMIs (A vs. B), down-DEMIs (A vs. C), and down-DEMIs (B vs. C). **(F)** Venn diagram shows two miRNAs that commonly existed in the comparison group of down-DEMIs (A vs. B), up-DEMIs (A vs. C), and up-DEMIs (B vs. C).

Total RNAs with good quality were obtained from 15 vitreous samples at an average concentration of 3.70 ± 5.01 ng per 20 µl of sample. In the sequencing data, the average raw tag counts detected from control, no-treatment group, and treatment group were 23,628,974, 19,404,071, and 24,028,428, respectively. After filtrating low-quality tags, the average clean tag counts of miRNA gained from each group were respectively 17,614,269, 16,870,205, and 20,184,021. Also, the average percentage of clean tags for all samples was 82.3%. Under the default local alignment settings, 43.5% clean tags were successfully aligned to reference genome performed by Bowtie2. Ultimately, a total of 405 known miRNAs matched to the miRbase database were identified, and 60 novel miRNAs were predicted by miRDeep2 (Supplementary Material: Table 2). Among the 465 miRNAs, 242, 317, and 334 miRNAs were respectively obtained from each group. A Venn graph was conducted to describe the distribution of miRNAs in each group (Figure 1A).

**TABLE 2:** Basic information of aflibercept-induced miRNA profile

Gene ID	Control expression	No-treatment expression	Treatment expression	log2 (control vs. no-treatment)	log2 (control vs. treatment)	log2 (no-treatment vs. treatment)
hsa-miR-204-3p	0	158.8	50.8	17.28	15.63	−1.64
hsa-miR-941	0.2	207.2	51.6	10.02	8.01	−2.01
hsa-miR-197-3p	1	665	61	9.38	5.93	−3.45
hsa-miR-3184-3p	0.6	282.6	136.8	8.88	7.83	−1.05
hsa-miR-181b-5p	1.2	323.4	59.2	8.07	5.62	−2.45
hsa-miR-30d-5p	3	568.8	155.8	7.57	5.7	−1.87
hsa-miR-3529-3p	3.6	427.2	63.6	6.89	4.14	−2.75
hsa-miR-24-3p	128.8	7276.2	342.8	5.82	1.41	−4.41
hsa-miR-3074-5p	92	4,661.6	1528.8	5.66	4.05	−1.61
hsa-miR-10a-5p	84.4	2343	604	4.79	2.84	−1.96
hsa-miR-196a-5p	20.8	574.6	64.8	4.79	1.64	−3.15
hsa-miR-338-5p	1.2	33	0.2	4.78	−2.58	−7.37
hsa-miR-27a-3p	56.6	1,472.8	146.8	4.7	1.37	−3.33
hsa-miR-532-5p	63	1,549.8	308.8	4.62	2.29	−2.33
hsa-miR-23a-3p	592.2	10,275	2263.2	4.12	1.93	−2.18
hsa-miR-28-3p	21.2	352.8	99	4.06	2.22	−1.83
hsa-let-7i-5p	575.6	7,153.2	2,769.6	3.64	2.27	−1.37
hsa-let-7d-3p	172.6	1,461.6	450.8	3.08	1.39	−1.7
hsa-miR-34a-5p	727.4	5,863	1,465.6	3.01	1.01	−2
hsa-let-7b-5p	1,006	7,414.6	3,345.4	2.88	1.73	−1.15
hsa-miR-222-3p	241	1,447.2	579.8	2.59	1.27	−1.32
hsa-miR-485-5p	25.2	102.8	0	2.03	−14.62	−16.65
hsa-miR-574-3p	21.6	59.8	0.6	1.47	−5.17	−6.64
hsa-miR-28-5p	17.6	43.4	0.2	1.3	−6.46	−7.76
hsa-miR-125b-5p	22.6	54.2	0.2	1.26	−6.82	−8.08
hsa-miR-19b-3p	2	0.6	53.8	−1.74	4.75	6.49
hsa-miR-143-3p	1,140	128	374	−3.15	−1.61	1.55
hsa-miR-7-5p	25	0.8	108	−4.97	2.11	7.08
hsa-miR-302d-3p	22.8	0	0.8	−14.48	−4.83	9.64

**FIGURE 2:**
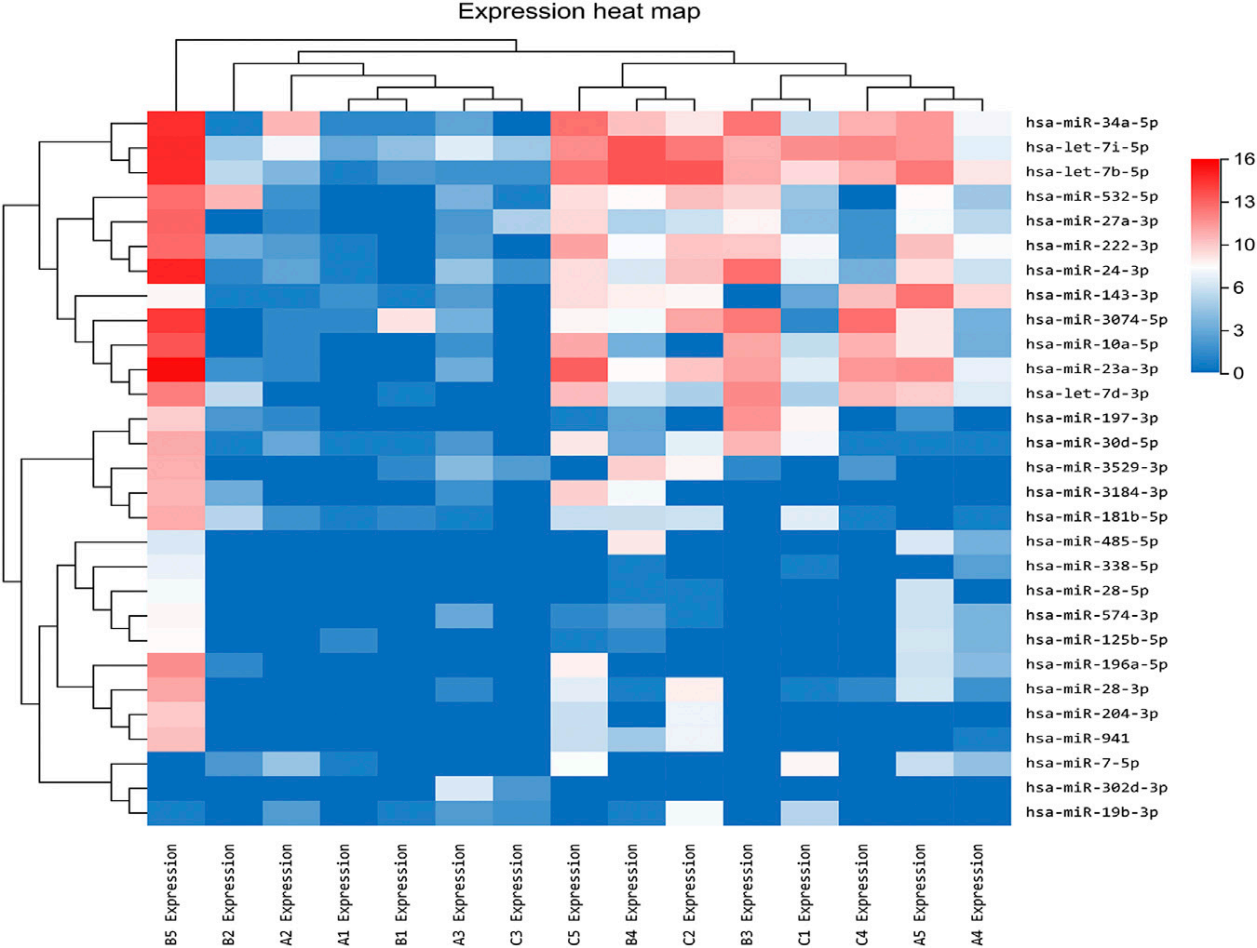
Aflibercept-induced miRNA profile visualized by hierarchical clustering. Red and blue respectively represent highly expressed and lowly expressed miRNAs. Sample A_1–5,_ B_1–5_, and C_1–5_ respectively represented five samples in control, no-treatment, and treatment groups.

In our previous research, a detailed analysis about miRNA profiles of control and no-treatment group has been described (Guo, Zhou et al., 2021). To further understand the complex regulation and expression of genes happening in the intraocular environment after intravitreal aflibercept injection, we selected and analyzed the differentially expressed miRNAs among three groups. In the comparison groups of no-treatment/control, treatment/control, and treatment/no-treatment, which were encoded as B/A, C/A, and C/B, respectively, 179 up-DEMIs and 46 down-DEMIs (B/A), 121 up-DEMIs and 51 down-DEMIs (C/A), and 73 up-DEMIs and 131 down-DEMIs (C/B) were screened out (shown in Figure 1B). In total, 29 miRNAs were selected as aflibercept-induced miRNAs, among which 20 miRNAs commonly existed in B/A up-DEMIs, C/A up-DEMIs, and C/B down-DEMIs (shown in Figure 1C); 2 miRNAs commonly existed in B/A down-DEMIs, C/A down-DEMIs, and C/B up-DEMIs (shown in Figure 1D); 5 miRNAs commonly existed in B/A up-DEMIs, C/A down-DEMIs, and C/B down-DEMIs (shown in Figure 1E); and 2 miRNAs commonly existed in B/A down-DEMIs, C/A up-DEMIs, and C/B up-DEMIs (shown in Figure 1F). Moreover, the expression of these 29 aflibercept-induced miRNAs in each group and even per sample is fully described in Figure 2 and Table 2. Samples A1–5, B1–5, and C1–5 in Figure 2 respectively represent five samples in control, no-treatment group, and treatment group.

### Function Analysis and Enrichment

**FIGURE 3:**
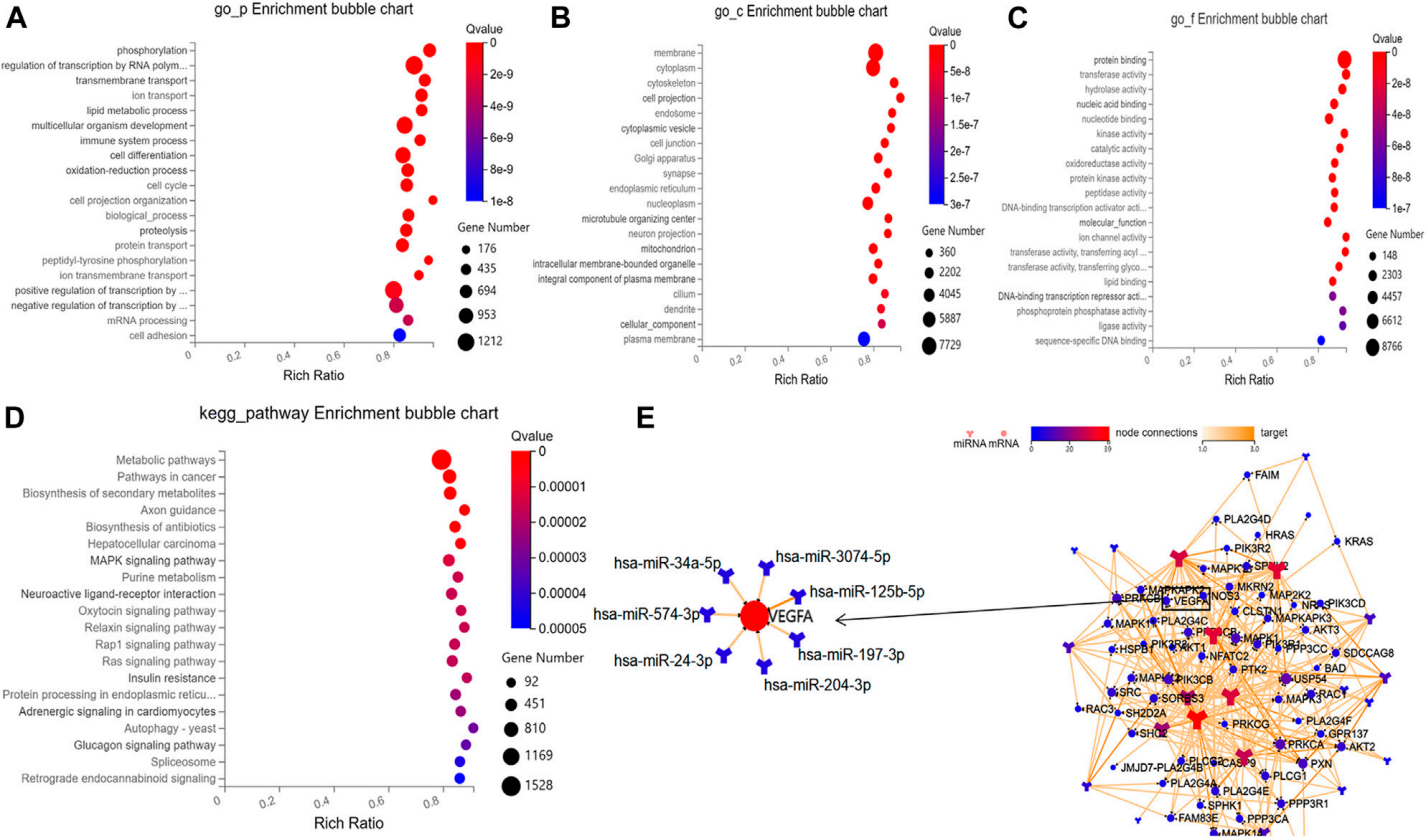
Results of bioinformatic analysis. **(A–C)** Bubble diagram respectively shows the top 20 GO enrichment annotations about biological process, cellular component, and molecular function of target mRNAs. **(D)** Bubble diagram exhibits the top 20 KEGG pathway analysis of target mRNAs. **(E)** The network illustrates the target relationship between the 29 aflibercept-induced miRNAs and mRNAs in VEGF signaling pathway, the specially marked parts of which show seven miRNAs that directly target VEGFA.

Based on the prediction results by RNAhybrid, Miranda, and TargetScan, the intersection of 129,936 human genes (including 19,984 mRNAs) were identified as putative targets of these aflibercept-induced miRNAs. To further gain insight into the molecular mechanisms underlying PDR, GO function and KEGG pathways enrichment analysis was performed in 19,984 target mRNAs. On the basis of the GO enrichment analysis, approximately 12,110 biological processes, 1,732 cellular components, and 4,233 molecular functions were identified with Q value (an adjusted *p*-value) <0.05 as the threshold, and the top 20 terms are shown in Figure 3A–C. The top listed GO terms related to cellular components (CC), molecular functions (MF), and biological process (BP) were respectively membrane (GO:0016020), protein binding (GO:0005515), and phosphorylation (GO:0016310). In the KEGG pathway analysis, approximately 392 pathways were enriched at Q value (adjusted *p*) <0.05, the top five of which were metabolic pathways (hsa01100; 1,528 mRNA), pathways in cancer (hsa05200; 604 mRNA), biosynthesis of secondary metabolites (hsa01110; 461 mRNA), axon guidance (hsa04360, 196 mRNA), and biosynthesis of antibiotics (hsa01130, 276 mRNA) (Figure 3D). What is more, 67 target mRNAs play roles in the VEGF signaling pathway with a Q value of 0.008 according to the enrichment result (hsa04370), and a network was conducted to illustrate the target relationship between the 29 miRNAs and mRNAs in VEGF signaling pathway (Figure 3E), the specially marked parts of which show seven miRNAs (hsa-miR-3074–5p, hsa-miR-125b-5p, hsa-miR-197-3p, hsa-miR-204-3p, hsa-miR-24-3p, hsa-miR-574-3p, hsa-miR-34a-5p) that directly target VEGFA.

### The Validation of Expression Profiles

**FIGURE 4:**
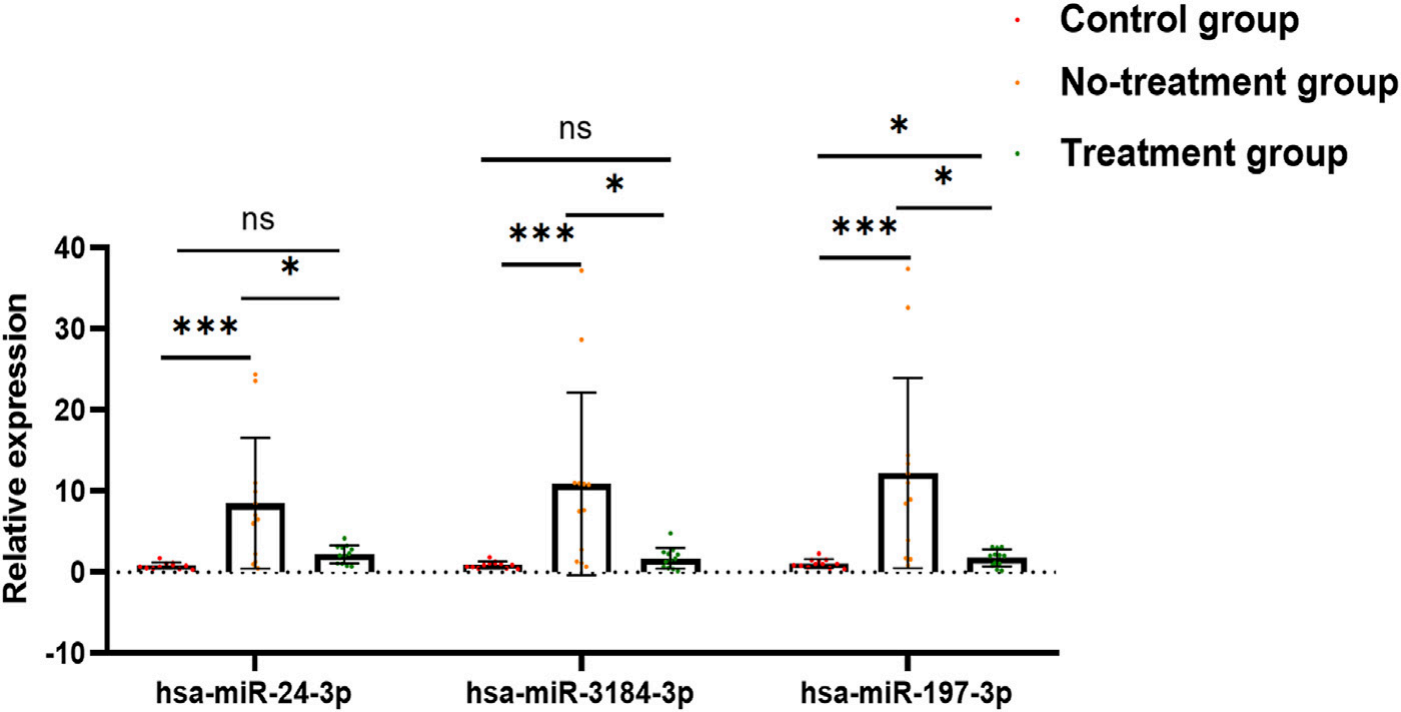
Bar graph exhibits the result of qRT-PCR. Has-miR-24-3p, hsa-miR-3184-3p, and hsa-miR-197-3p were significantly upregulated in no-treatment group compared with control group, and significantly downregulated in treatment group compared with no-treatment group. There was no significant difference in the expression of has-miR-24-3p and hsa-miR-3184-3p between control group and treatment group (*p* = 0.078, *p* = 0.543). Hsa-miR-197-3p was upregulated in treatment group compared to control group. *p < 0.05, **p < 0.01, ***p < 0.001, ns: no significance.

After a comprehensive analysis, three miRNAs were randomly selected among the 29 aflibercept-induced miRNAs to confirm the expression profiles. The validation result was listed in Supplementary File S1, Table 3. As expected, the qRT-PCR result was consistent with the NGS result, indicating the reliability of the expression profiles (Figure 4; Table 3). In the validation cohort, hsa-miR-3184-3p, hsa-miR-24-3p, and hsa-miR-197-3p were significantly upregulated in the no-treatment group compared with the control group, and significantly downregulated in the treatment group compared with the no-treatment group (*p* < 0.05). There was no significant difference in the expression of has-miR-24-3p and hsa-miR-3184-3p between control group and treatment group (*p* = 0.078, *p* = 0.543). Hsa-miR-197-3p was upregulated in the treatment group compared with the control group.

**TABLE 3:** QPCR verification of aflibercept-induced miRNA profile

Gene ID	Sequence	Control expression (25%, 75%)	No-treatment expression (25%, 75%)	Treatment expression (25%, 75%)	H	*p*
hsa-miR-197-3p	UGG​CUC​AGU​UCA​GCA​GGA​ACA​G	0.65, 1.45	2.29, 14.18	1.08, 3.12	17.437	<0.001
hsa-miR-3184-3p	AAA​GUC​UCG​CUC​UCU​GCC​CCU​CA	0.52, 1.10	1.68, 11.00	0.58, 2.43	15.176	0.001
hsa-miR-24-3p	UUC​ACC​ACC​UUC​UCC​ACC​CAG​C	0.49, 1.08	1.51.10.74	0.91, 2.81	14.375	0.001

**FIGURE 5:**
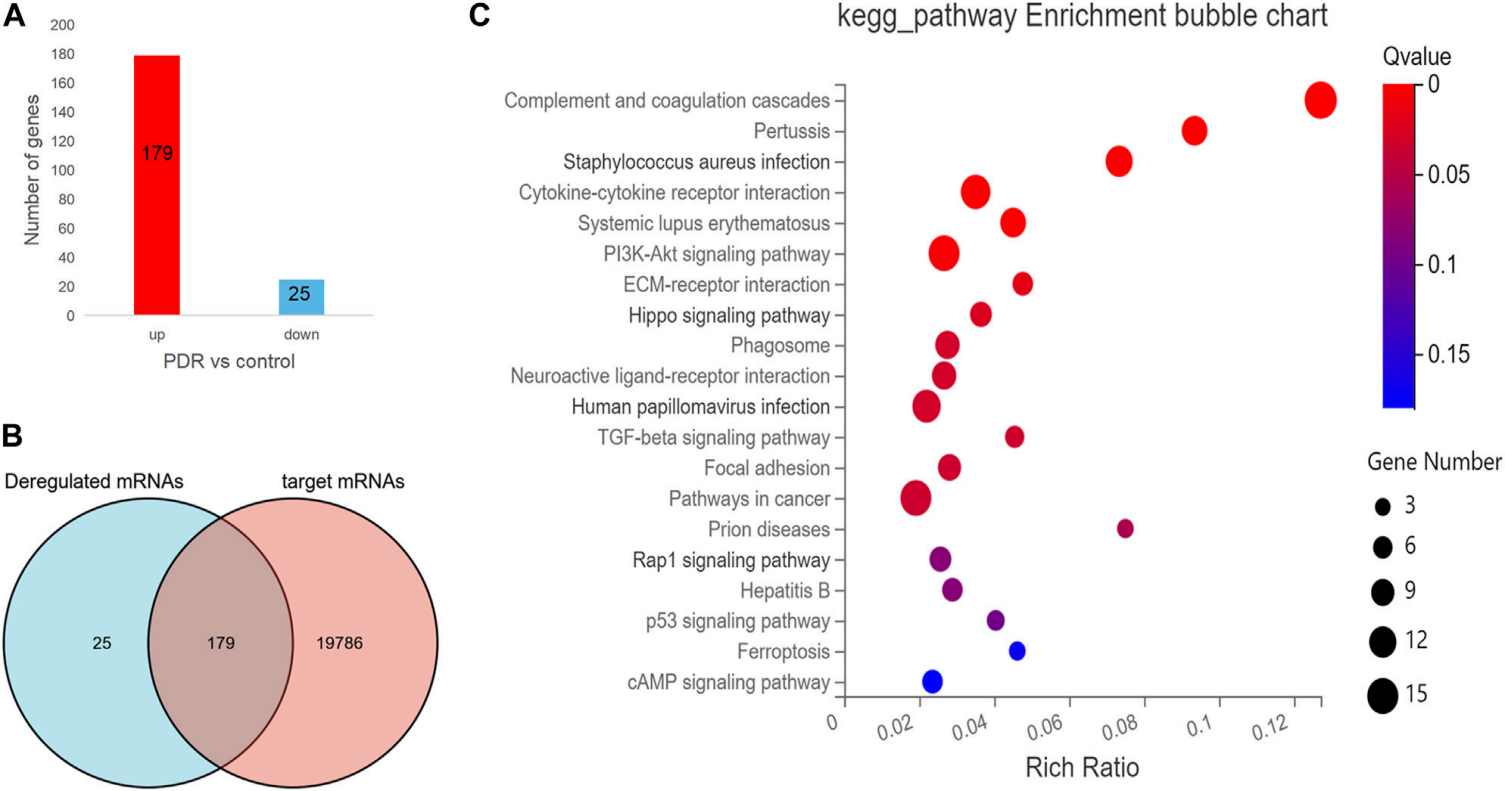
The analysis for the protein-coding mRNA profile in GSE160310 data. **(A)** Bar graph in red visualizes 179 upregulated protein-coding mRNAs in PDR patients compared with controls; bar graph in yellow represents 25 downregulated protein-coding mRNAs in PDR patients compared with controls. **(B)** Venn diagram describes the 179 overlapped mRNAs between the 19,984 target mRNAs in our experiment and 204 deregulated protein coding mRNAs in the GSE160310 profiling data. **(C)** Bubble diagram exhibits the top 20 KEGG pathway analyses of 179 overlapped mRNAs.

To further understand the expression of 19,984 target mRNAs in PDR patients, the raw data (GSE160310) about microarray expression profiling of total RNA in retinal tissues of human post-mortem donors were retrieved from the GEO database, and the data of 20 healthy controls and 5 PDR donors were included in this study. In the new profiling result, 204 protein-coding mRNAs were found to be differentially expressed in the PDR group compared with healthy control, including 179 upregulated and 25 downregulated mRNAs (Figure 5A), and finally we only identified 179 overlapped mRNAs between the 19,984 target mRNAs in our experiment and 204 deregulated protein-coding mRNAs in the GSE160310 profiling data (Figure 5B). The KEGG pathway annotation for 179 overlapped mRNAs is shown in Figure 5C, and it revealed that immunity (complement and coagulation cascades, hsa04610), infections (pertussis, hsa05133; *Staphylococcus aureus* infection, hsa05105), inflammation (cytokine–cytokine receptor interaction, hsa05322), immune diseases (systemic lupus erythematosus fiber, hsa05322), formation of fibrous tissue (TGF-beta signaling pathway, hsa04350), cell growth, and death-related pathways (p53 signaling pathway, hsa04115) were highly involved.

## Discussion

Recently, the miRNA profile in the vitreous from PDR patients compared with non-diabetes mellitus controls has been detected by different sequencing technologies in few studies (Mammadzada et al., 2019; Martinez and Peplow 2019; Martins, Amorim et al., 2020). In a recent study, it has been demonstrated that miR-20a-5p, miR-20a-3p, miR-20b, miR-106a-5p, miR-27a-5p, miR-27b-3p, miR-206-3p, and miR-381-3p were dysregulated in the retina and serum of diabetic mice, by which the expressions of VEGF, BDNF, PPAR-α, and CREB1 were modified (Platania et al., 2019). Moreover, the modulation of miRNA expression has also been evaluated in the serum of diabetic retinopathy (DR) patients (Trotta et al., 2021). In another study, authors assessed the expression of miRNAs in the vitreous humor of patients diagnosed with retinal detachment and different grading of proliferative vitreoretinopathy (Toro et al., 2020). In the present study, we used NGS to analyze the altered miRNA profiles in the vitreous from patients with PDR induced by anti-VEGF (aflibercept) therapy. Twenty-nine miRNAs were identified to be significant aflibercept-induced miRNA profiles in vitreous of patients with PDR. In another cohort, hsa-miR-3184-3p, hsa-miR-24-3p, and hsa-miR-197-3p were randomly selected from the aflibercept-induced miRNA profile to validate the expression by qRT-PCR, and the results showed that they were significantly upregulated in the no-treatment group compared with non-diabetic controls, and significantly downregulated in the treatment group compared with the no-treatment group, indicating good reliability and reproducibility of the sequencing analysis.

To elucidate possible functional roles of these aflibercept-induced miRNAs, 129,936 human genes (including 19,984 mRNAs) were predicted as putative targets of DEMIs. On the basis of the GO enrichment analysis for 19,984 target mRNAs, phosphorylation (biological processes), membrane (cellular components), and protein binding (molecular functions) were listed as top GO terms. A previous study indicated that protein phosphorylation was closely related to the pathogenesis of diabetes (Dey, Hao et al., 2017). In the KEGG pathway analysis, the top five terms were metabolic pathways, pathways in cancer, biosynthesis of secondary metabolites, axon guidance, and biosynthesis of antibiotics. A recent study demonstrated that anti-VEGF therapy increases glycolytic metabolites (Keunen, Johansson et al., 2011). In class-3 semaphorin (SEMA) and members of neuropilins (NRPs) and plexins (PLXNs), the major roles in axon guidance pathway have been reported to show a positive correlation with increased cell sensitivity or resistance to anti-angiogenesis drug treatments (Maione, Capano et al., 2012; Zhang, Shao et al., 2020). In rat models of retinopathy of prematurity, the mRNA expression of VEGF164 and SEMA3A was demonstrated to be elevated at the early age (Loporchio, et al., 2021). In the present study, hsa-miR-204-3p was predicted to commonly target SEMA3A, VEGF-A, NRP1, and PLXNA2, and the target relation between PLXNA2 and hsa-miR-204-3p has been confirmed by dual-luciferase reporter gene assay in another research (Chi et al., 2009). Thus, intravitreal aflibercept injection may increase some drug response–related mRNA levels by regulating the expression of hsa-miR-204-3p.

Among 19,984 target mRNAs, 67 mRNAs play roles in the VEGF signaling pathway, including VEGF-A. What is more, seven miRNAs (hsa-miR-3074-5p, hsa-miR-125b-5p, hsa-miR-197-3p, hsa-miR-204-3p, hsa-miR-24-3p, hsa-miR-574-3p, hsa-miR-34a-5p) were predicted to directly target VEGF-A. By reviewing literatures, the target relations between VEGF-A and hsa-miR-34a-5p (Ye et al., 2008) were verified using dual-luciferase reporter gene assay. Dong et al., through *in vitro* and *in vivo* analysis, demonstrated that increased miR-34a-5p significantly decreased VEGF-A mRNA level, whereas the decrease was partially restored when TUG1 level was increased in hepatoblastoma (Dong, Liu et al., 2016). However, in this study, hsa-miR-34a-5p was highly expressed in PDR patients and significantly downregulated in PDR patients after anti-VEGF therapy. We suspect it is due to the expression of VEGF-A that may be influenced by a number of factors, and anti-VEGF therapy induced the lower expressed hsa-miR-34a-5p, which has a potential trend toward increased VEGF-A mRNA level. It means that intravitreal aflibercept injection may induce the expression of VEGF-A; in other words, hsa-miR-34a-5p may associate with the resistance to anti-VEGF treatment. Nevertheless, a lot of research needs to be performed to further investigate this hypothesis.

In the validation cohort, the expression of hsa-miR-3184-3p, hsa-miR-24-3p, and hsa-miR-197-3p were confirmed to be upregulated in PDR patients, and anti-VEGF therapy significantly downregulated their expression. Based on previous literature, miR-24-3p has also been demonstrated to be rich in diabetic exosome, which induces a highly limited mRNA expression of phosphatidylinositol 3-kinase regulatory subunit gamma (PIK3R3), and the reduction of PIK3R3 inhibited human umbilical vein endothelial cell (HUVEC) proliferation, migration, and angiogenesis, as well as inducing HUVEC apoptosis (Xu et al., 2020). Another research has a similar finding—extremely high miR-24-3p level in exosomes was derived from microglial cells that were injected into the vitreous of oxygen-induced retinopathy (OIR) mice, and it showed that exosome-treated OIR mice exhibited smaller avascular areas and fewer neovascular tufts in addition to decreased VEGF and transforming growth factor β (TGF-β) expression (Xu et al., 2019). In a cohort of 40 patients with type 1 diabetes mellitus, the doubling of hsa-miR-197-3p and hsa-miR-24-3p level corresponded to a sixfold higher stimulated C-peptide level and 4.2% lower insulin dose–adjusted HbA1c value, respectively (Samandari et al., 2017). Therefore, intravitreal aflibercept injection may involve in regulating angiogenesis through reduced expression of hsa-miR-24-3p.

Finally, 179 overlapped mRNAs between the 19,984 target mRNAs in our experiment and 204 deregulated protein-coding mRNAs in the GSE160310 profiling data were included for further analysis. According to KEGG pathway annotation, the top five terms were complements and coagulation cascade pathways, pertussis pathway, *Staphylococcus aureus* infection pathway, cytokine–cytokine receptor interaction pathway, and systemic lupus erythematosus pathway. The complement and coagulation cascade pathways have been reported to contribute to the regulation of hepatic fibrosis and liver cirrhosis caused by excessive extracellular matrix deposition (Huang et al., 2021). In this investigation, C1QA and C3, the main recognition proteins in complement and coagulation cascade pathways, pertussis pathway, *S. aureus* infection pathway, and systemic lupus erythematosus pathway, were highly expressed in PDR samples. Elevated C1QA level may increase transcriptional activation of the classical complement pathway via the formation of C3 and C5 convertases, so that it can induce increased susceptibility to complement-mediated liver damage and fibrosis (Donat et al., 2020; Huang et al., 2021). In our prediction, the target relation existed in hsa-miR-24-3p and C1QA, providing an important point to verify. Cytokine–cytokine receptor interaction pathway may play roles in the regulation of angiogenesis, leukocyte development, tumor growth, and metastasis (Charo and Ransohoff 2006). In addition, TGF-β signaling pathway, another critical pathway in PDR, has been frequently reported to play roles in retinal fibrosis, retinal detachments, and choroidal neovascularization (Raju et al., 2013; Bonfiglio et al., 2020). In brief, the regulation of angiogenesis and retinal fibrosis was the most possible explanation for the function analysis of 179 overlapped mRNAs in this investigation.

Nevertheless, there are some limitations in the present study. First, the bioinformatic analysis results need further verification *via in vitro* and *in vivo* experiments, especially on the target relation between miRNAs and critical mRNAs. Second, our study was limited to the analysis of the miRNA profile in the three groups, and we did not include the observation of the therapeutic effect in their follow-up. What is more, the results need to be further substantiated in a large cohort study.

## Conclusion

In conclusion, we provided the miRNA expression profile of five IMH patients, five PDR patients with no history of treatment, and five PDR patients who were at the seventh day after anti-VEGF therapy using NGS to realize the biochemical activities occurring in the ocular environment from the transcriptional level. Twenty-nine dysregulated miRNAs were identified as aflibercept-induced miRNAs, of which the expression of hsa-miR-3184-3p, hsa-miR-24-3p, and hsa-miR-197-3p was validated in another cohort. Bioinformatic analysis and literature study revealed several roles of aflibercept-induced miRNAs and alternation of drug sensitivity or resistance-related mRNAs, regulating some critical mRNAs involved in angiogenesis and retinal fibrosis. However, further verification *via in vitro* and *in vivo* experiments is required to explore the mechanism.

## Data Availability

The original contributions presented in the study are included in the article/Supplementary Material, further inquiries can be directed to the corresponding authors.

## Footnotes

1
https://61.49.19.26/login

2
http://bowtie-bio.sourceforge.net/index.shtml

3
https://omictools.com/cmsearch-tool

4
https://github.com/rajewsky-lab/mirdeep2

5
https://bibiserv.cebitec.unibielefeld.de/rnahybrid

6
http://www.microrna.org/micro-rna/home.do

7
http://www.targetscan.org/vert_72/

8
http://bioinfo.au.tsinghua.edu.cn/software/degseq/

9
https://www.ncbi.nlm.nih.gov/geo/
